# Comment on “A rare case of 9 years congenital muscular torticollis treated with complete unipolar sternocleidomastoid release: A case report and literature review”

**DOI:** 10.1016/j.ijscr.2022.107586

**Published:** 2022-08-31

**Authors:** Minaam Farooq, Shah Gul Zahra, Mohammad Ashraf, Mahnoor Humayun

**Affiliations:** a Department of Neurosurgery, King Edward Medical University, Mayo Hospital, Lahore, Pakistan; b Department of Neurosurgery, Allama Iqbal Medical College, Jinnah Hospital Lahore, Pakistan and Wolfson School of Medicine, University of Glasgow, Scotland, United Kingdom; c Dow Medical College, Karachi, Pakistan

**Keywords:** Congenital, Torticollis, Case report, Unipolar release

Dear Editor,

An amazing case report was recently published by Kurniawan et al. under the title “A rare case of 9 years congenital muscular torticollis treated with complete unipolar sternocleidomastoid release: A case report and literature review” [1]. Congenital muscular torticollis refers to a neck deformity that primarily involves a shortened sternocleidomastoid muscle (SCM) resulting in the patient's head turning toward the affected ipsilateral side and the chin pointing contra-laterally [2]. With the help of detailed history, examination and relevant investigations such as X-ray cervical spine and MRI cervical spine, we rule out the other causes of torticollis before the patient is marked as the case of congenital torticollis. When diagnosed early, torticollis can be managed in an excellent manner using conservative physiotherapy [2]. However, if surgery is required, optimal time for it is between 1 and 4 years as stated by Ling et al. [3]. Unipolar SCM release, bipolar SCM release with or without Z plasty, selective denervation and dorsal cord stimulation are the surgical approaches used for the treatment of congenital muscular torticollis. We did the similar study on 28 adolescent patients of neglected congenital muscular torticollis [4]. Our article was submitted for publication 3 months back and got published about one and a half month ago. Since our study was in the submission process, the authors of existing case report couldn't cite our article which seems to be very similar to theirs. So, herein we describe some points that resemble and enrich this case report.

1. Patients in our study, like this case report, underwent unipolar release of sternocleidomastoid muscle from the sternoclavicular end and the range of neck motion was assessed. Similarly, patients were recommended three months of manual stretching after being discharged from the hospital. Additionally, a cervical collar for three months was also advised to patients, which we believe can improve the range of neck motion.
2. Kurniawan et al. reported full cervical range of motion by doing assessment using the cervical mandibular angle (CMA) on the follow up of their patient. Similar to this, the outcome in our series was also assessed using the cervical mandibular angle (CMA). Additionally, we used an adapted version of the modified Lee's criteria to assess the outcomes [5].
3. Our study, in resemblance to this case report, supports that unipolar release is more efficient and safer than bipolar release for the treatment of congenital muscular torticollis. We also appreciate and support Kurniawan et al. for the fact that unipolar release should be preferred over extensive surgical option like bipolar release as facial and spinal accessory nerves at the mastoid end would be at risk in it.

## Funding

There is no funding.

## Ethical approval

Not applicable for this study.

## Consent

Not applicable for this study.

## Author contribution

Minaam Farooq contributed in review of the literature and editing.

Shah Gul Zahra and Mohammad Ashraf contributed to writing the draft and revision.

## Registration of research studies

Not applicable.

## Guarantor

Not applicable for this study.

## Declaration of competing interest

There are no conflicts of interest.
